# The crystal structure of Zika virus helicase: basis for antiviral drug design

**DOI:** 10.1007/s13238-016-0275-4

**Published:** 2016-05-12

**Authors:** Hongliang Tian, Xiaoyun Ji, Xiaoyun Yang, Wei Xie, Kailin Yang, Cheng Chen, Chen Wu, Heng Chi, Zhongyu Mu, Zefang Wang, Haitao Yang

**Affiliations:** School of Life Sciences, Tianjin University, Tianjin, 300072 China; Tianjin International Joint Academy of Biotechnology and Medicine, Tianjin, 300457 China; The State Key Laboratory of Pharmaceutical Biotechnology, School of Life Sciences, Nanjing University, Nanjing, Jiangsu 210023 China; Cleveland Clinic Lerner College of Medicine of Case Western Reserve University, Cleveland, OH 44195 USA

**Dear Editor**

The genus of *Flavivirus* contains important human pathogens, including dengue (DENV), yellow fever (YFV), West Nile (WNV), Japanese encephalitis (JEV), and tick-borne encephalitis (TBEV) viruses, which cause a number of serious human diseases throughout the world (Pierson TC, 2013). Zika virus (ZIKV) is also an arthropod-borne flavivirus, which was initially isolated in 1947 from a febrile sentinel rhesus monkey in the Zika forest in Entebbe, Uganda. ZIKV is transmitted by multiple *Aedes* mosquitoes (Lazear and Diamond, 2016). Historically, ZIKV infection typically caused a mild and self-limiting illness in human beings, accompanied by fever, headache, arthralgia, myalgia, and maculopapular rash (Ioos et al., 2014). ZIKV caught global attention in April 2007, when it caused a large epidemic of Asian genotype ZIKV in Yap Island and Guam, Micronesia. From 2013 to 2014, the Asian genotype was found responsible for the epidemics among several Pacific Islands, including French Polynesia, New Caledonia, Cook Islands, Tahiti, and Easter Island (Lazear and Diamond, 2016). In 2015, a rampant outbreak of ZIKV infection struck Brazil and other regions of the Americas, causing an estimated 1.3 million cases (Hennessey et al., 2016; Mlakar et al., 2016). Thereafter, ZIKV was found in fetal brain tissue, presumably accounting for the sharp increase of congenital microcephaly in the epidemic areas (Brasil et al., 2016; Mlakar et al., 2016; Rodrigues, 2016). Recent studies have demonstrated the significant cellular death of neural stem cells once infected with ZIKV, which provides direct evidence for the inhibitory role of ZIKV on fetal brain development (Tang et al., 2016). However, as there are currently no effective vaccines or therapies available to contain ZIKV infection, ZIKV remains a significant challenge to the public health of the Western Hemisphere as well as the whole world (Lazear and Diamond, 2016).

Similar to other flaviviruses, ZIKV contains a single-stranded, positive sense RNA genome of 10.7 kb. The genome is translated into a single large polypeptide, which undergoes proteolytic cleavage into 3 structural proteins (C, prM/M, and E), and 7 non-structural proteins (NS1, NS2A, NS2B, NS3, NS4A, NS4B, and NS5) (Pierson TC, 2013). The NS3 protein is a key component for viral polypeptide processing and genomic replication, with a protease domain at its N-terminus and a helicase domain at the C-terminus. Upon stimulation by RNA, the helicase domain exhibits intrinsic nucleoside triphosphatase activity, which then provides the chemical energy to unwind viral RNA replication intermediates to facilitate replication of the viral genome together with RNA-dependent RNA polymerase (NS5) (Lindenbach, 2001). Given its essential role in genome replication, ZIKV helicase could be an attractive target for drug development against ZIKV.

Here we report the crystal structure of ZIKV helicase at 1.8-Å resolution. The helicase structure revealed a conserved triphosphate pocket critical for nonspecific hydrolysis of nucleoside triphosphates across multiple flavivirus species. A positive-charged tunnel has been identified in the viral helicase, which is potentially responsible for accommodating the RNA. This crystal structure of ZIKV helicase provides an accurate model for rational drug design against ZIKV infection.

We determined the crystal structure of ZIKV helicase at a resolution of 1.8 Å (Table S1) in the space group *P2*_*1*_. Distinct from the DENV-2 helicase, whose two crystal forms both contain two molecules per asymmetric unit (Xu et al., 2005), ZIKV helicase has a solo protein molecule in an asymmetric unit in the crystals. No stable oligomer through crystallographic packing was identified in the crystals, consistent with the observation of a monomeric form of the ZIKV helicase in solution by size exclusion chromatography (Fig. 1A). This observation suggests that ZIKV helicase is able to function as a monomer. The refined model is complete and includes the residues 175–617 from ZIKV NS3. Although the overall structure is generally well ordered, the electron densities are less well defined for residues 193–202 and 249–255 with a higher B factor (>50 compared with an overall average B factor of 27). This indicates that these are possible substrate/ligand binding regions due to the increased flexibility. The tertiary structure of ZIKV helicase reveals three domains, of around 130–160 amino acid residues each (Fig. 1B and 1C). Domain I (residues 175–332) and domain II (residues 333–481) share a similar fold with an expanded six-stranded β-sheet stacked between a large number of loops and four helices, though there is little sequence identity between these two domains. Domain III (residues 482–617) is predominantly comprised of a four-α-helix bundle broadened by two antiparallel β strands partially exposed to the solvent. The three domains are well distinguished by clear clefts. Two α-helices from domain I interact with the approximately parallel α-helix bundle from domain III. Domain II associates with domain III via a long β-hairpin standing at the back of the molecule. The featured motifs of the superfamily 2 helicases (Caruthers and McKay, 2002), functionally coupled with NTP hydrolysis and nucleic acid binding making them attractive drug targets, exist in domains I and II and map to interdomain clefts.

**Fig. 1:**
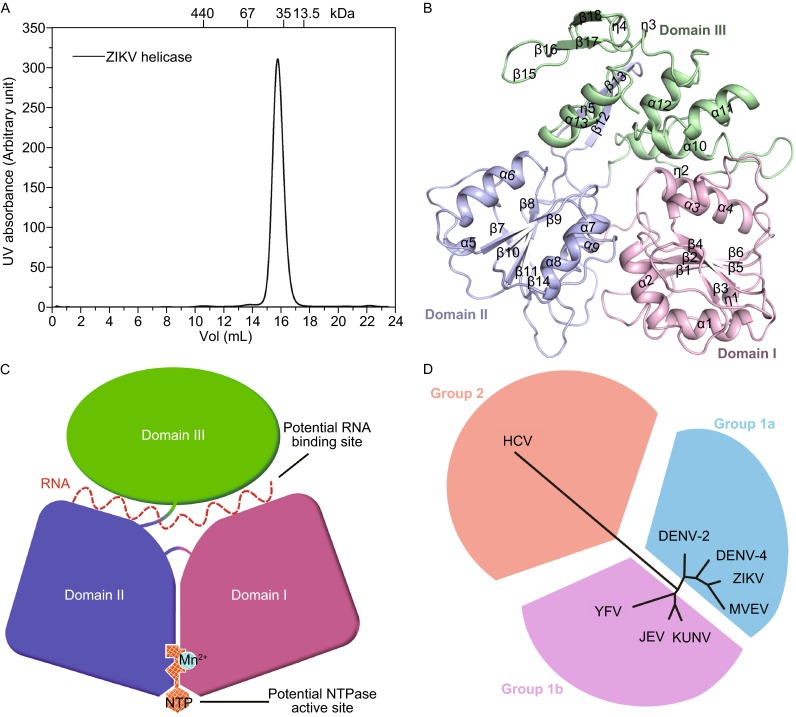
The monomeric structure of ZIKV helicase. (A) Size-exclusion chromatograms of ZIKV helicase. The molecular masses of protein standards are indicated at the top. (B) The overall structure of ZIKV helicase with the three domains colored and labeled respectively. (C) A cartoon diagram illustrating of the overall fold with potential RNA binding site and NTPase active site labelled. (D) Structure-based phylogenetic tree of 8 viral helicase structures from the *Flaviviradae* family using the program SHP (Stuart et al., 1979) and PHYLIP (Felsenstein, 1997). The following structures with PDB ID in parentheses are included: DENV-2 (2BMF), DENV-4 (2JLQ), JEV (2Z83), KUNV (2QEQ), YFV (1YKS), MVEV (2V8O), HCV (1HEI)

ZIKV helicase is evolutionarily close to those from Murray Valley encephalitis virus (MVEV), DENV-4, DENV-2, YFV, JEV, Kunjin virus (KUNV), and Hepatitis C virus (HCV) from the *Flaviviradae* family, whose structures have already been solved. To gain further structural insight, we generated a structure-based phylogenetic tree for these homologous helicases (Fig. 1D), using the Structure Homology Program (Stuart et al., 1979). Structural superposition of these 8 structures reveals that all of the flavivirus helicases, including the helicase of ZIKV, cluster into one large group (Group 1), while HCV helicase falls into a separate one (Group 2). In Group 1, ZIKV helicase is evolutionarily closer to those of MVEV, DENV-4 and DENV-2 (Group 1a) while the other members cluster into Group 1b. Clustering of viral helicases indicate that they share more structural features, suggesting it might be possible to design wide-spectrum inhibitors against all the group/subgroup members.

The NTPase active site is located in the cleft between Domain I and II (Fig. 2A). In this cleft, Walker A and B motifs (motifs I and II, respectively) (Fig. S1) play an important role in recognizing NTP and cations (Mn^2+^ or Mg^2+^) (Caruthers and McKay, 2002). A network of solvent molecules is also buried in this pocket. Since the structure of ZIKV helicase in complex with NTP and the cation is currently unavailable, the structure of AMPPNP-Mn^2+^ bound to DENV-4 helicase, which is a close homologue to ZIKV helicase, serves as a good model for analysis (Fig. 2B) (Luo et al., 2008). Residues K200, T201, R202 (motif I, also called P-loop, Fig. 2C), D285, E286 (motif II), Q455, R459, and R462 (motif VI) of ZIKV helicase superimposed well on their counterparts in DENV-4 helicase: K199, T200, K201 (P-loop), D284, E285 (motif II), Q456, R460, and R463 (motif VI) of DENV-4 helicase, respectively (Fig. 2D). Based on structural homology, these residues of ZIKV helicase are likely to play similar roles in NTP hydrolysis. For instance, the side chain of K200 could be responsible for interacting with the γ-phosphate of the nucleotide during transition state stabilization; the strictly conserved D285 and E286 residues could participate in coordinating the divalent cation. In the overlaid structures, the base and ribose groups of AMPPNP bulge out from the binding pocket, implying that ZIKV helicase would lack nucleotide specificity for its NTPase activity.

**Fig. 2:**
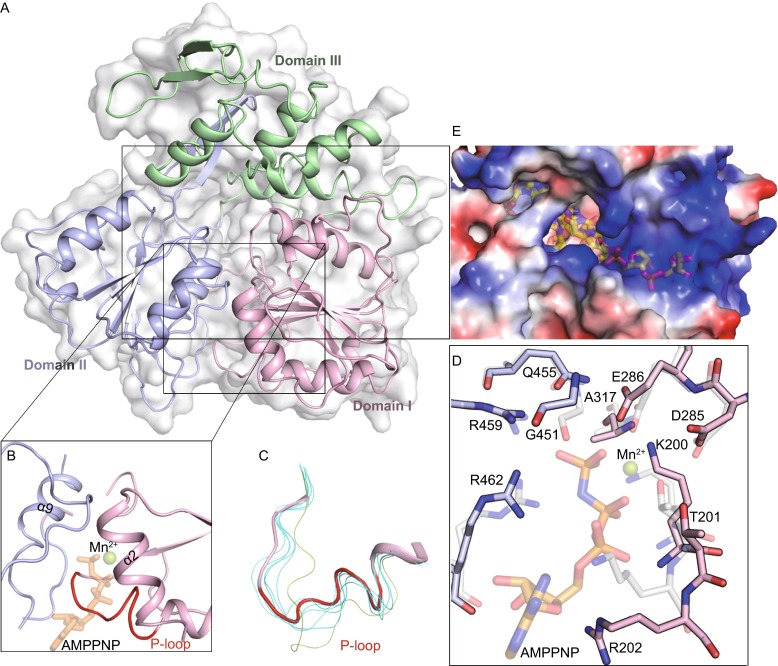
Structural insight into ZIKV helicase. (A) Cartoon and surface representation of the overall fold with the three domains of ZIKV helicase, colored and labeled respectively; (B) The electrostatic surface representation showing the tunnel for potential RNA binding. Positive potentials are colored blue and the negative are colored red. The putative position of the nucleic acid is marked as semi-transparent sticks. The model was obtained by superposition with the DENV-4 helicase in complex with ssRNA (PDB code 2JLV). (C) A clear view of the NTPase active site. The positions of putative nucleotide substrate (as sticks) and Mn^2+^ (as sphere) are marked semi-transparently by superposition with the DENV-4 helicase bound to AMPPNP and Mn^2+^ (PDB code 2JLR). P-loop is shown in red. (D) Isolated  P-loops are shown by superimposing the structures of 7 flavivirus apo helicases. ZIKV helicase is in red ribbon and the others are shown in finer lines. The P-loop of DENV-4 helicase is colored green. The following structures of helicases with PDB ID in parentheses are included: DENV-2 (2BMF), DENV-4 (2JLQ), JEV (2Z83), KUNV (2QEQ), YFV (1YKS), MVEV (2V8O). (E) Interactions at NTPase active site by superposition of ZIKV helicase (solid) with DENV-4 helicase in complex with AMPPNP and Mn^2+^ (semitransparent, PDB code 2JLR). Conserved residues are shown as sticks and labeled

It is worthwhile to note that the P-loop, which is critical for NTP binding and catalysis, has a variety of structural conformations among flavivirus helicases (Fig. 2C), even though the amino acid sequences are stringently conserved. This discrepancy highlights the high degree of intrinsic flexibility of the P-loop. Interestingly, the conformation of the P-loop in ZIKV helicase (apo form) is quite similar to that of DENV-4 helicase complexed with AMPPNP-Mn^2+^, which is however, distinct from the conformation in its own apo form. This implies that ZIKV helicase might not need to undergo as significant local rearrangement of the NTP binding pocket to transition into the active state as in DENV-4 helicase.

In the structure of ZIKV helicase, a positively charged tunnel can be clearly identified along the domain boundary of Domain III, which directly interacts with Domain I and Domain II (Fig. 2E). The tunnel is lined with positively charged residues and remains wide enough to accommodate a single strand (ss) nucleic acid in an extended conformation running through Domain II to Domain I. The positively charged residues, most of which were contributed by Domain I and Domain II, presumably stabilize the sugar-phosphate backbone of the nucleic acid. Superposition of ZIKV helicase to DENV-4 helicase bound with a 12-mer ssRNA (PDB ID 2JLV) (Luo et al., 2008) generated a model to analyze the potential pattern for nucleic acid binding. It seems that rearrangement of the three domains is required to build a non-clashing model of ZIKV helicase for RNA binding, which has been seen in DENV-4 helicase bound to RNA. Interestingly, P363, P233, D409, and T264, which contribute specificity of DENV-4 helicase for RNA, are entirely conserved in ZIKV helicase, thus implying that ZIKV helicase would prefer RNA to DNA.

In summary, the recent outbreak of ZIKV and its association with fetal abnormalities have caused global public health emergency. Here we present a high-resolution structure of ZIKV helicase, which is an important drug target. The structure has revealed critical substrate-binding pockets for antiviral drug design. Pharmaceutical development of inhibitors targeting the RNA binding tunnel and the pivotal regulatory regions would be a plausible strategy for innovative anti-ZIKV therapies.

## FOOTNOTES

We would like to thank Zuokun Lu for data collection at beamline BL18U1 of the Shanghai Synchrotron Radiation Facility (SSRF); Erin Weber and Lanfeng Wang for discussion and advice. This work was supported by the National Basic Research Program (973 Program) (Nos. 2015CB859800 and 2014CB542800) and the National Natural Science Foundation of China (Grant No. 31528006).

Haitao Yang and Hongliang Tian conceived and designed the experiments. Hongliang Tian, Xiaoyun Yang, Wei Xie, Heng Chi and Zhongyu Mu performed the experiments. Haitao Yang, Xiaoyun Ji, Cheng Chen, Chen Wu and Zefang Wang analyzed the data. Haitao Yang, Hongliang Tian, Xiaoyun Ji and Kailin Yang wrote the paper. Hongliang Tian, Xiaoyun Ji, Xiaoyun Yang, Wei Xie, Kailin Yang, Cheng Chen, Chen Wu, Heng Chi, Zhongyu Mu, Zefang Wang, and Haitao Yang declare that they have no conflict of interest. This article does not contain any studies with human or animal subjects performed by the any of the authors.

## Electronic supplementary material

Below is the link to the electronic supplementary material.

Supplementary material 1 (PDF 1224 kb)
