# Supplementary material for: The crystal structure of Zika virus helicase: basis for antiviral drug design
Source: Protein Cell. 2016 May 12;7(6):450–4. doi: 10.1007/s13238-016-0275-4 (PMC4887331; doi:10.1007/s13238-016-0275-4)
Supplement: Supplementary file 1 — Supplementary material 1 (PDF 1224 kb) [file 13238_2016_275_MOESM1_ESM.pdf]

SUPPLEMENTAL MATERIALS

Cloning and expression

The catalytic domain of the ZIKV helicase (residues 172 to 617) was amplified by PCR using the forward primer 5'-CGCGGATCCGAAGAGACACCGGTGGAGT-3' and the reverse primer 5'-CCGCTCGAGTTACCGTTTTCCGGCTGCGAA-3'. The underlined regions correspond to *Bam*HI and *Xho*I sites, respectively. The coding sequence for ZIKV helicase was cloned into the vector pET.32M.3C and fused at its N-terminus to thioredoxin and a $(His)_6$ tag followed by PreScission Protease (GE) cleavage site. Transformed *Escherichia coli* BL21 (DE3) clones were grown in LB medium at 37°C and then induced by 0.2 mM isopropyl-ß-D-thiogalactopyranoside at 16°C. After overnight growth, cells were harvested via centrifugation.

Protein purification and crystallization

Cells resuspended in lysis buffer A (20 mM Na_2HPO_4, pH 8.0, 0.5 M NaCl and 20 mM imidazole) were lysed by high pressure homogenization and the lysate was clarified by centrifugation at 30,000×g for 40 min at 4°C. The supernatant was purified by Ni Sepharose (GE) affinity chromatography equilibrated with buffer A. Proteins were eluted using buffer A supplemented with 250 mM imidazole. After concentration by ultrafiltration and dilution in buffer B (20 mM Na_2HPO_4, pH 8.0, 0.5 M NaCl), the fraction containing Trx-$(His)_6$-ZIKV helicase was cleaved with PreScission Protease at 4°C for approximately 12 h. The cleavage mixture was loaded onto a HiTrap Q 5 mL column (GE) pre-equilibrated with buffer C (50 mM Tris-HCl,

pH 8.0, 50 mM NaCl) and eluted using a linear NaCl concentration gradient. Concentrated protein of interest was subjected to a final gel-filtration purification step through a HiLoad 16/600 Superdex 200^{TM} PG column (GE) in buffer D (10 mM Tris-HCl, pH 8.0, 150 mM NaCl, 5mM dithiothreitol, and 5% glycerol). Crystals of native ZIKV helicase (stored in buffer D) were grown at 18°C by the microbatch-under-oil method and the crystallization condition consisted of 0.1 M Sodium citrate tribasic dihydrate pH 5.5, 22% (w/v) polyethylene glycol 1000. A volume of 1 μL of precipitating solution was mixed with an equal volume of ZIKV helicase at a concentration of 5 mg mL^{-1}.

Crystal data collection, structure determination and refinement

Crystals were cryoprotected using the crystallization buffer with 30% glycerol and flash-frozen in liquid nitrogen. Diffraction data were collected at 100 K at Shanghai Synchrotron Radiation Facility (SSRF) beamline BL18U1 with wavelength of 0.97791 Å. Diffraction data were processed using HKL3000 (Minor, 1997) in space group $P2_1$. The data statistics are summarized in Supplementary Table 1. The structure was solved by molecular replacement using the structure of DENV-2 helicase (PDB ID 2BHR) as a search model. The program PHASER (McCoy et al., 2007) was used for the molecular replacement search. The initial model was auto-built by Buccaneer (Cowtan, 2006) and refined through iterative rounds of TLS and restrained refinement using Refmac5 (Murshudov et al., 2011), followed by rebuilding manually using Coot (Emsley and Cowtan, 2004). Final refinement was finished by PHENIX (Afonine et al., 2012). The final model has an R_{work}/R_{free} of

18.0%/20.0%. The refinement statistics are summarized in Supplementary Table 1. The refined coordinates have been deposited in the PDB under accession number 5JMT.

Structural analysis and illustrations

Pairwise superposition of a series of helicase structures was performed using the program SHP (Stuart et al., 1979). A full matrix of evolutionary distances was calculated and the tree representation was generated using the program PHYLIP (Felsenstein, 1997).

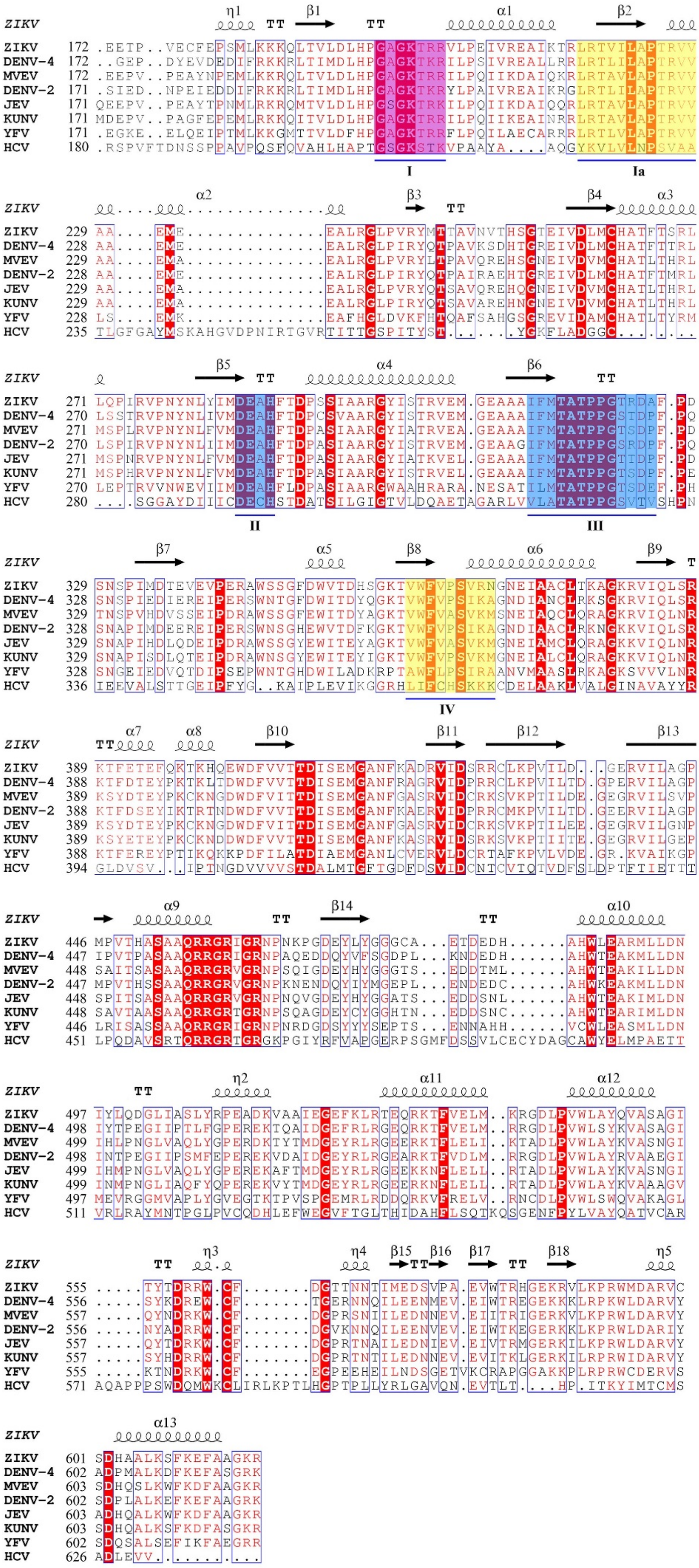
ZIKV
DENV-4
MVEV
DENV-2
JEV
KUNV
YFV
HCV
I
Ia
II
III
IV

Supplementary Figure S1. Structural alignment of *Flaviviridae* helicases by ESPript (Robert and Gouet, 2014).

The sequences of ZIKV (accession no. KU312312), DENV-4 (accession no. AY618990), MVEV (accession no. AF161266), DENV-2 (accession no. AY037116), JEV (accession no. M55506), KUNV (accession no. AY274504), YFV (accession no. AF052437) and HCV (accession no. AF009606), were obtained from GenBank. Secondary structure elements of ZIKV helicase are displayed above the sequence alignment. The conserved motifs among superfamily 2 helicases are indicated.

Supplementary Table S1. Data collection, phasing and refinement statistics

	ZIKV helicase
**Data Collection**	
X-ray Source	SSRF beamline BL18U1
Wavelength (Å)	0.97791 Å
Space group	$P2_1$
Unit cell a, b, c, α, β, γ (Å, °)	53.4, 69.0, 57.9, 90.0, 94.4, 90.0
Resolution range* (Å)	50.0-1.80 (1.83-1.80)
Unique reflections	37570 (1351)
Completeness (%)	96.2 (69.5)
Redundancy	6.2 (3.3)
$I/\sigma I$	22.7 (3.0)
R_{merge}	0.083 (0.365)
**Refinement**	
Resolution range (Å)	44.3-1.80
No. of reflections (working/test)	35650/1877
R_{work}/R_{free}	0.180/0.200
Number of atoms	
Protein	3516
Water	439
B-factors	
Protein	27.1
Water	35.7
r.m.s. deviations	
Bond lengths (Å)	0.003
Bond angles (°)	0.777

* Numbers in the brackets are for the highest resolution shell.

SUPPLEMENTAL REFERENCES

Afonine PV, Grosse-Kunstleve RW, Echols N, Headd JJ, Moriarty NW, Mustyakimov M, Terwilliger TC, Urzhumtsev A, Zwart PH, Adams PD (2012) Towards automated crystallographic structure refinement with phenix.refine. Acta Crystallogr D Biol Crystallogr 68:352-367

Cowtan K (2006) The Buccaneer software for automated model building. 1. Tracing protein chains. Acta Crystallogr D Biol Crystallogr 62:1002-1011

Emsley P, Cowtan K (2004) Coot: model-building tools for molecular graphics. Acta Crystallogr D Biol Crystallogr 60:2126-2132

Felsenstein J (1997) An alternating least squares approach to inferring phylogenies from pairwise distances. Syst Biol 46:101-111

McCoy AJ, Grosse-Kunstleve RW, Adams PD, Winn MD, Storoni LC, Read RJ (2007) Phaser

crystallographic software. J Appl Crystallogr 40:658-674
Minor W, Otwinowski Z (1997) Processing of X-Ray Diffraction Data Collected in Oscillation Mode. Methods in Enzymology 276: Macromolecular Crystallography, part A, 307-326
Murshudov GN, Skubak P, Lebedev AA, Pannu NS, Steiner RA, Nicholls RA, Winn MD, Long F, Vagin AA (2011) REFMAC5 for the refinement of macromolecular crystal structures. Acta Crystallogr D Biol Crystallogr 67:355-367
Robert X, Gouet P (2014) Deciphering key features in protein structures with the new ENDscript server. Nucleic Acids Res 42:320-324
Stuart DI, Levine M, Muirhead H, Stammers DK (1979) Crystal structure of cat muscle pyruvate kinase at a resolution of 2.6 Å. J Mol Biol 134:109-142
